# Enzalutamide-Resistant Progression of Castration-Resistant Prostate Cancer Is Driven via the JAK2/STAT1-Dependent Pathway

**DOI:** 10.3389/fmolb.2021.652443

**Published:** 2021-10-22

**Authors:** Yong Luo, Xiaobing Yang, Spyridon P. Basourakos, Xuemei Zuo, Dechao Wei, Jiahui Zhao, Mingchuan Li, Qiankun Li, Tao Feng, Pengju Guo, Yongguang Jiang

**Affiliations:** ^1^ Department of Urology, Beijing Anzhen Hospital, Capital Medical University, Beijing, China; ^2^ Department of Genitourinary, The University of Texas MD Anderson Cancer Center, Houston, TX, United States

**Keywords:** castration-resistant prostate cancer, CXCR7, JAK2, STAT1, enzalutamide, resistance

## Abstract

Previous studies showed that CXCR7 expression was upregulated after enzalutamide (ENZ) treatment, and an increased level of CXCR7 could increase the invasion, migration, and angiogenesis of castration-resistant prostate cancer (CRPC) cells. This study demonstrated that the levels of p-JAK2, p-STAT1, C-Myc, and VEGFR2 were significantly reduced after CCX771, a specific CXCR7 inhibitor, treatment. This effect further increased after the combination treatment of ENZ and CCX771. Then, we verified that targeting the inhibition of JAK2 or STAT1 could remarkably increase apoptosis and DNA damage and decrease the migration of CRPC cells. More importantly, the combination treatment of ENZ + JAK2/STAT1 led to much greater suppression than the single-agent treatment of JAK2 or STAT1. Subcutaneous CRPC xenograft tumor growth was also reduced by single-agent ENZ treatment and single-agent FLUD, a specific STAT1 antagonist, treatment; but much superior effect was elicited by the combination treatment of ENZ + FLUD. The proliferative indices significantly decreased following combination treatment in tumor tissues compared with control-treatment tissues and single-agent-treatment tissues. Our results demonstrated that CXCR7, which signifies an androgen receptor (AR)-independent signaling pathway, caused CRPC progression via the downstream JAK2/STAT1 signal transduction cascade. Combined inhibition targeting both the AR and JAK2/STAT1 resulted in substantial tumor suppression due to the reduction in DNA damage repair ability and increment in apoptosis.

## Introduction

Castration-resistant prostate cancer (CRPC) is an extremely advanced state of prostate cancer progression, which indicates a complete failure of traditional androgen deprivation therapy (ADT) and has a very inadequate prognosis. ENZ, a second-generation androgen receptor (AR) antagonist, is an important treatment option for patients with CRPC, which significantly prolongs the median overall survival of CRPC to 32.4 months (Beer et al., 2014). However, about 42% of patients with CRPC are not sensitive to ENZ treatment (Azuma, 2018). Even if the treatment is effective, ENZ resistance (Beer et al., 2014) usually occurs after 11.2 months. This modest survival benefit is related to a significant variation in the rate of development of resistance pathways among individuals in clinical trials. It also indicates the need for a deeper understanding of the molecular mechanisms that lead to androgen ablation resistance and the development of more effective predictive biomarkers and therapeutic approaches for CRPC. Therefore, how to overcome ENZ resistance has become a critical research focus in this field.

Extensive research suggests that, among several risk factors, inflammation plays an important role in the development and progression of primary PCa to metastatic disease (Carver et al., 2011). Elucidating the details of inflammatory signaling pathways not only promotes the understanding of the tumor progression mechanism but also helps confirm the molecular characteristics of cases vulnerable to immunotherapy. Additionally, chemokines and chemokine receptor networks are fundamental components of the complex interactions between inflammatory cells and PCa cells. The key essence of ENZ resistance is the process in which AR activity is usually suppressed by drugs; meanwhile, the AR bypass pathways are continuously activated. Our previous study (Luo et al., 2018) confirmed that CXCR7 derepression was closely associated with ENZ resistance. When AR activity was inhibited by ENZ, CXCR7 began to derepress from AR suppression and further drove CRPC cell progression through increasing the potency of anti-apoptosis, rapid proliferation, DNA repair, and angiogenesis. After combination with the CXCR7 antagonist (CCX771), the ENZ treatment response was significantly improved in both CRPC cellular and animal models. Li also demonstrated that elevated CXCR7 expression activated MAPK/ERK signaling through ligand-independent, but β-arrestin 2–dependent, mechanisms and led to tumor progression upon enzalutamide resistance. The inhibition of MAPK/ERK activation could remarkably suppress ENZ-resistant CRPC (Li et al., 2019). Rafiei found that CXCR7 derepression induced by macrophage migration inhibitory factor could upregulate the expression of cell cycle genes through activating the Akt signaling pathway and drive cellular ENZ-resistant growth (Rafiei et al., 2019). More recent reports confirmed that CXCR7 derepression was closely involved in the androgen ablation–resistant process. However, the underlying mechanism of how CXCR7 triggers CRPC progression and survival remains unclear.

The Janus kinase/signal transducer and activator of transcription (JAK/STAT) signal transduction pathway is a master signaling pathway that always regulates cell survival, apoptosis, hypertrophy, and collagen synthesis in the heart of mammals and is activated in response to oxidative stress, inflammatory cytokines, and growth factors. The JAK/STAT signaling pathway plays an important role in various physiological processes of cell proliferation, differentiation, tumorigenesis, immune function, and hematopoiesis (Bolli et al., 2003). The present study verified that CXCR7 derepression drove CRPC cell progression and migration via the JAK2/STAT1 pathway. It also reported the synergistic effect of ENZ plus specific JAK/STAT inhibitor to inhibit the survival, invasion, and migration of CRPC cells and suppress the growth of CRPC xenograft tumors.

## Materials and Methods

### Cell Line and Reagents

The human androgen-independent prostate cancer cell line C4-2B (American Type Culture Collection, VA, United States) was validated by short tandem repeat DNA fingerprinting with an AmpFLSTR Identifiler Kit (Applied Biosystems, CA, United States) at MD Anderson’s Characterized Cell Line Core Facility. The cells were cultured in DMEM containing 1 mM sodium pyruvate, 2.5 mM glutamine, 10% FBS, 100 U/ml penicillin, and 100 μg/ml streptomycin at 37°C in a humidified incubator containing 5% CO_2_. The serum-free medium was regularly used in the cell transfection trials. Enzalutamide (AR inhibitor), AMD3100 (CXCR4 inhibitor), and AZD1480 (ATP competitive antagonist of JAK2) were purchased from SelleckChem, and SDF-1 (CXCR4/7 activated ligand) was purchased from GenScript. CCX771 (CXCR7-specific inhibitor) and CCX704 (the control inhibitor of CXCR7, with the molecular structure similar to CCX771 but not binding to CXCR7) were provided by ChemoCentryx (CA, United States). Fludarabine (STAT1 synthesis inhibitor) and GSI-XII (Notch-1 inhibitor) were obtained from MedChemExpress. In addition, C4-2B cells were seeded at desired densities (5 × 10^5^/well in a six-well plate for western blotting and wound-healing assay; 1 × 10^5^/well in a 24-well plate for flow cytometry; and 1.5 × 10^4^/well in a six-well plate for ELISA).

### *In Vitro* ENZ and CCX771 Treatment

C4-2B cells were pretreated with 100 ng/ml SDF-1 + 1 M ENZ or 100 ng/ml SDF-1 + 800 nM CCX771 for 24 h. On the following day, the cells were again treated with 20 μg/ml AMD3100, 25 µM GSI-XII, or 1 µM CCX704 for 48 h. Additionally, the cells in the ENZ and CCX771 groups were treated with a single drug for 72 h. After treatment, the cellular protein was extracted for western blotting to assess the downstream factors of the CXCR7 signaling pathway.

### *In Vitro* JAK2 and STAT1 Inhibition Treatment

C4-2B cells were transfected with siRNA to knock down JAK2 or STAT1 and further cultured for 72 h. Meanwhile, the cells were treated with 5 µM AZD1480 or 5 µM fludarabine for 72 h to inhibit the activity of JAK2 and STAT1, respectively. After treatment, cellular extraction was used for western blotting to assess the downstream factors of the JAK2/STAT1 pathway.

### *In Vitro* Combined Inhibition of JAK2/STAT1 and AR or CXCR7

C4-2B cells were transfected with siRNA for 24 h. On the following day, the cells were further treated with vehicle control DMSO, 100 ng/ml SDF-1 + 800 nM CCX771, or 100 ng/ml SDF-1 + 1 µM ENZ for 48 h. After treatment, all samples were comparatively evaluated for the change profile of apoptosis, migration, and DNA damage.

### siRNA and Plasmid Transfection

The cells were seeded 1 day before siRNA transfection. JAK2 (clone ID: OHu24049, GenScript), the STAT1 (clone ID: OHu15845, GenScript) plasmid, and the control vector were transfected into cells using X-tremeGENE HP DNA transfection reagent (Roche). All samples were prepared 72 h after plasmid transfection for western blotting, wound-healing assay, flow cytometry analysis, and ELISA.

### Western Blotting

Clarified protein lysates were separated on denaturing SDS-PAGE and electro-transferred onto nitrocellulose membranes. The membranes were initially incubated with 5% non-fat dry milk in TBS for 2 h and then probed at 4°C overnight with the following antibodies: anti-CXCR7 (ab138509, 1:250), anti-DNA-PKCs (ab44815, 1:500), anti-VEGFR2 (ab39638, 1:50) (Abcam, MA, United States), anti-JAK2 (cs4040, 1:1,000), anti-P-JAK2 (cs4406, Tyr1007, 1:500), anti-STAT1 (cs9176, 1:1,000), anti-P-STAT1 (cs8826, Ser727, 1:500), anti-C-Myc (cs9402, 1:1,000), anti-cleaved-PARP-1 (cs5625, 1:500), anti-Bcl-2 (cs4223, 1:1,000), anti-γH2AX (cs7631, 1:250) (Cell Signaling, MA, United States), and anti-GAPDH (sc47724, 1:1,000) (Santa Cruz Biotechnology, CA, United States). The membranes were then hybridized with horseradish peroxidase (HRP)-conjugated secondary antibody (sc2004/2005, Santa Cruz Biotechnology) for 2 h at room temperature. An enhanced chemiluminescence system (AMRESCO, Inc., OH, United States) was used to detect the immunopositive protein bands. Additionally, the cell lysis buffer containing phosphatase inhibitors (Na_3_VO_4_/1 mM) was regularly applied during phosphorylated protein extraction and detection. Each experiment was repeated five times.

### Flow Cytometry

The cells were harvested and stained with propidium iodide for 30 min at room temperature. The analysis was performed at 405 nm excitation and emission with a 450/50 band-pass filter using a FACSCanto II Flow Cytometer (BD Biosciences, NJ, United States). The histograms of DNA content were analyzed using the FlowJo software (Tree Star, Inc., OR, United States) to determine cell cycle distribution. Each experiment was repeated three times.

### Wound-Healing Assay

The cells were grown to 80% confluence in six-well plates in RPMI with 10% FBS and antibiotics. A straight scratch was made using a 10 µl pipette tip at three different sites (top, middle, and bottom) in triplicate experiments. The medium was removed, and the cells were washed with the culture medium to remove the floating cell debris. After treatment, the cells were fixed and stained with crystal violet dye. Wound closure was measured using MRI Wound Healing Tool macro for ImageJ (v1.50b). Each experiment was repeated three times.

### DNA Fragmentation Assay

A DNA fragmentation assay was performed for apoptotic evaluation following the protocol of the Cell Death Detection ELISA Kit. Each experiment was repeated three times.

### Subcutaneous C4-2B Xenografts

The cells (1 × 10^6^) were resuspended in serum-free DMEM, mixed 1:1 with Matrigel (BD Biosciences, NJ, United States), and then injected subcutaneously into the left flanks of previously castrated SCID mice (Charles River Laboratories, MA, United States). When palpable tumors reached a volume of 30–50 mm^3^, the mice were randomly divided into different experimental groups to receive one of the following treatments for 28 days: DMSO, ENZ (10 mg/kg, oral gavage, daily), fludarabine (1 mg/kg, IP, 5 days/2 weeks), and ENZ + fludarabine. Tumor size was monitored by measuring two dimensions, and the volume was calculated as length × width^2^/2. When the 28-day protocol was finished, all mice were euthanized by carbon dioxide inhalation, and the tumors were harvested. The CO_2_ displaced the euthanized chamber at the rate of 10–30% of chamber volume per minute. The mice were also euthanized ahead of the protocol if they became severely weak or if the tumor size reached 20 mm.

### Immunohistochemistry

Ki67 immunohistochemistry was carried out on formalin-fixed and paraffin-embedded tissue sections from the 28-day C4-2B xenografts after *in vivo* treatment. After tissue sections were deparaffinized and rehydrated through graded alcohol, they were heated in a microwave in 0.01 mol/L citrate buffer at pH 6.0 for 10 min to retrieve antigens. After a 30 min incubation in Dako protein blockage solution, the tissue sections were incubated in rabbit monoclonal antibody against Ki67 (1:300; Santa Cruz Biotechnology) for 90 min, followed by incubation in an HRP polymer−conjugated secondary antibody (Dako, Glostrup, Denmark) for 40 min. The immunoreaction was visualized in DAB/H_2_O_2_. The specificity of immunoreactions was verified by replacing the primary antibodies with PBS. Ten high-power fields were selected randomly in slides of each group, and the numbers of positively stained cells were counted and the percentage of positive cells was calculated.

### Statistical Analysis

Data were presented as mean ± standard deviation and analyzed with ANOVA with Tukey’s post hoc test. Statistical analysis was undertaken using SPSS 13.0 for Windows (SPSS, Inc., IL, United States). Two-sided *p* values < 0.05 indicated a statistically significant difference.

## Results

### Inhibition of CXCR7 Activity Decreased the Phosphorylation of JAK2/STAT1

**FIGURE 1:**
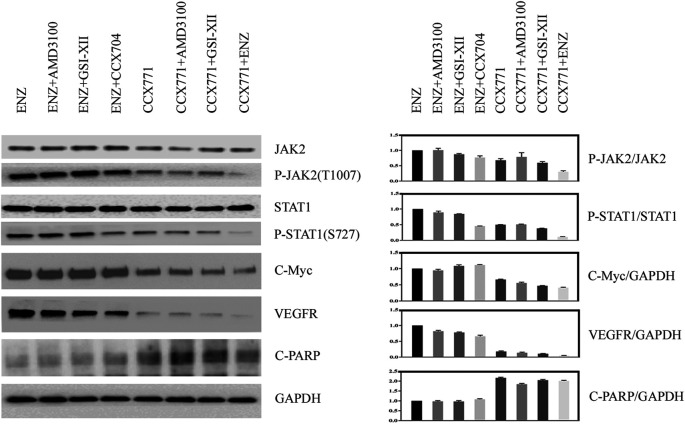
Comparison of CXCR7 downstream signaling proteins between CCX771-based treatment and ENZ-based treatment. **(A)** Western blotting demonstrated that the expression of phosphorylated JAK2, phosphorylated STAT1, C-Myc, and VEGFR markedly decreased, but the expression of cleaved-PARP-1 increased in CCX771-based treatment cells. The combination treatment of CCX771 + ENZ exhibited much greater synergistic suppression of phosphorylated JAK2, phosphorylated STAT1, C-Myc, and VEGFR. **(B)** All western blot band densities were quantitatively analyzed.

To explain how combination treatment of ENZ + CCX771 inhibited CRPC development, we detected some critical proteins of the JAK/STAT signaling pathway in C4-2B cells under different treatment conditions. Western blotting revealed a possible signaling mechanism of combined treatment. As shown in Figure 1, we demonstrated that the phosphorylation of JAK2 and STAT1 could be reduced significantly by CCX771-based treatment than ENZ-based treatment in C4-2B cells. In addition, the expression levels of C-Myc, an important proliferative regulator, and VEGFR, a critical angiogenesis producer, also markedly decreased in CCX771-based treatment cells, but the expression of cleaved-PARP-1 increased in CCX771-based treatment cells. More interestingly, the addition of ENZ to CCX771-treated cells (combination treatment group) exhibited much greater synergistic suppression of phosphorylated JAK2, phosphorylated STAT1, C-Myc, and VEGFR. ENZ treatment−induced CXCR7 upregulation was verified as the activator of the JAK2/STAT1 signaling pathway.

### Inhibition of JAK2/STAT1 Activity Led to Cellular DNA Damage and Apoptosis

**FIGURE 2:**
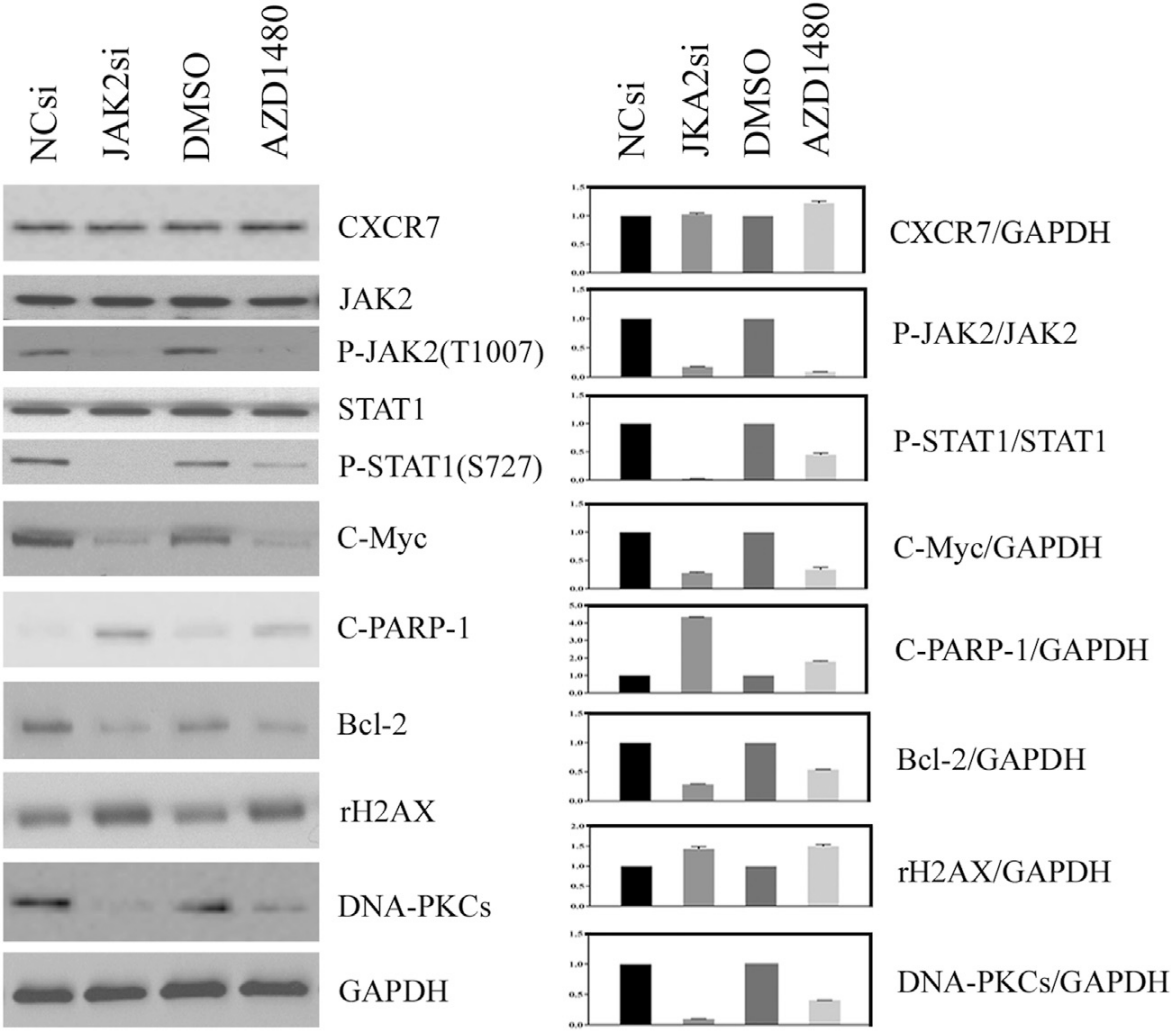
Change profile of downstream functional proteins after the inhibition of JAK2 activity. **(A)** Western blotting revealed that, after knocking down the expression of JAK2 by siRNA transfection and repressing the activity of JAK2 by AZD1480 in C4-2B cells, the expression of CXCR7 did not display any change, but the expression of p-STAT1, oncogene C-Myc, and anti-apoptotic protein Bcl-2 decreased significantly. In addition, the expression of DNA damage marker γH2AX increased, the expression of non-homologous end-joining **(NHEJ)** repairing protein DNA-PKCs significantly decreased, but the expression of homologous repairing **(HR)** protein C-PARP-1 increased. **(B)** All WB band densities were quantitatively analyzed.

**FIGURE 3:**
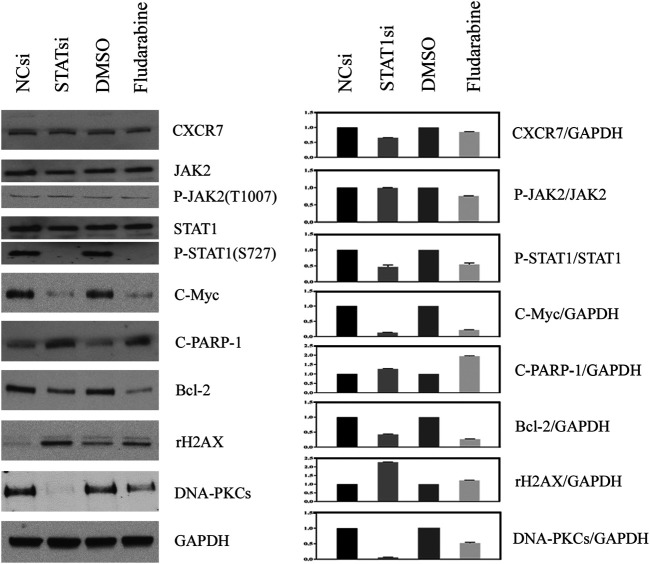
Change profile of downstream functional proteins after the inhibition of STAT1 activity. **(A)** Western blotting revealed that, after knocking down the expression of JAK2 by siRNA transfection and repressing the activity of JAK2 by FLUD in C4-2B cells, the expression of CXCR7 and P-JAK2 did not display any change, but the expression of C-Myc and Bcl-2 decreased remarkably. In contrast, the expression of DNA damage marker γH2AX increased apparently, the expression of homologous repairing **(HR)** protein C-PARP-1 increased accordingly, but the expression of non-homologous end-joining **(NHEJ)** repairing protein DNA-PKCs decreased measurably. **(B)** All WB band densities were quantitative analyzed.

The effect of JAK2/STAT1 activity on the expression of downstream functional proteins was confirmed by western blotting. After knocking down the expression of JAK2 by siRNA transfection and repressing the activity of JAK2 with AZD1480 in C4-2B cells, the expression of CXCR7 did not display any change, but the expression of p-STAT1 decreased significantly. In addition, the expression of oncogene C-Myc and anti-apoptotic protein Bcl-2 apparently decreased, while the expression of DNA damage marker γH2AX increased. Moreover, the expression of non-homologous end-joining (NHEJ) repairing protein DNA-PKCs significantly decreased, but the expression of homologous repairing (HR) protein C-PARP-1 increased (Figure 2). Similarly, the inhibition of STAT1 activity did not affect the expression of CXCR7 and phosphorylated JAK2. The expression of C-Myc, Bcl-2, and DNA-PKCs remarkably decreased, while the expression of γH2AX and C-PARP-1 significantly increased (Figure 3).

### ENZ + JAK2/STAT1si Synergistically Suppressed the Survival, Migration, and DNA Damage Repair of CRPC Cells

**FIGURE 4:**
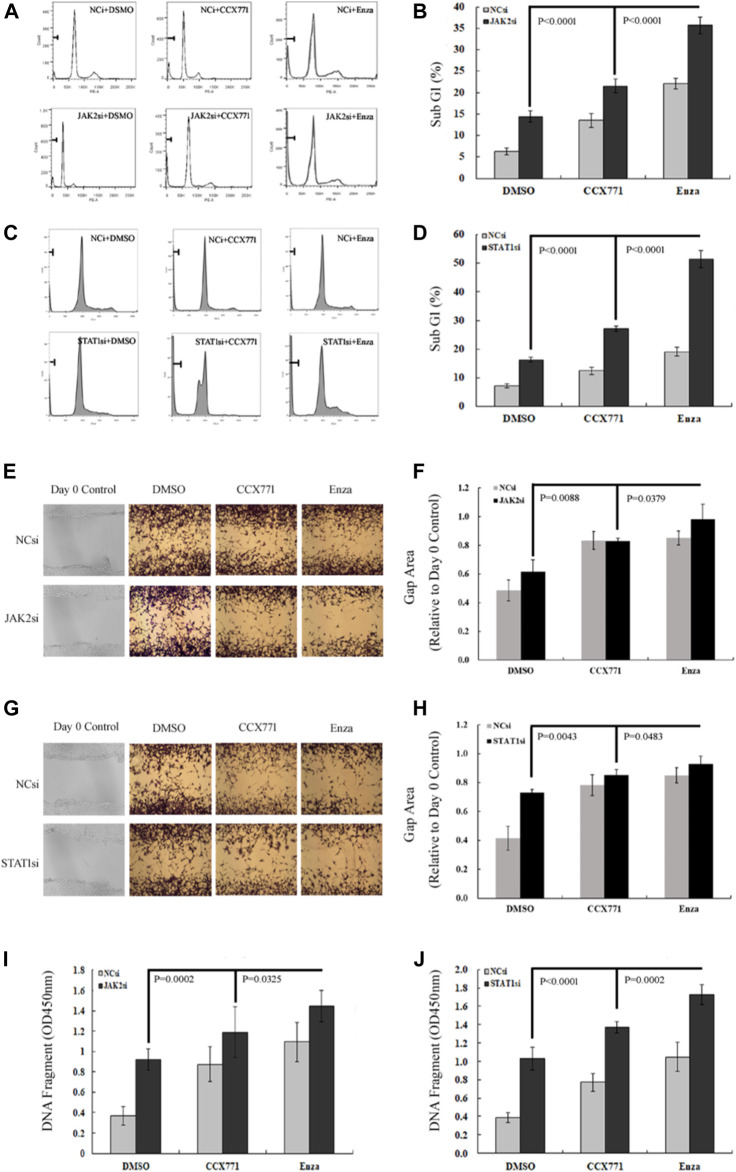
ENZ + JAK2/STAT1si synergistically suppressed survival, migration, and DNA damage repair of CRPC cells. **(A,B)** Flow cytometry demonstrated that single-agent JAK2si treatment remarkably increased the percentage of sub-G1 cells, and the combination treatment of ENZ + JAK2si showed a significantly superior effect compared with single-agent JAK2si treatment and the combination treatment of CCX771 + JAK2si. **(C,D)** Similarly, STAT1si significantly increased the percentage of sub-G1 cells, and the combination treatment of ENZ + STAT1si also showed a much greater effect compared with single-agent STAT1si treatment and the combination treatment of CCX771 + STAT1si. **(E,F)** Wound-healing assay results showed that the combination treatment of ENZ + JAK2si could reduce migration compared with single-agent JAK2si treatment and the combination treatment of CCX771 + JAK2si. **(G,H)** Wound-healing assay results showed that the combination treatment of ENZ + STAT1si also reduced migration compared with single-agent STAT1si treatment and the combination treatment of CCX771 + STAT1si. **(I)** ELISA results showed that the combination treatment of ENZ + JAK2si exhibited much stronger ability to induce DNA damage compared with single-agent JAK2si treatment and the combination treatment of CCX771 + JAK2si. **(J)** Similarly, the combination treatment of ENZ + STAT1si also led to much apparent DNA damage compared with single-agent STAT1si treatment and the combination treatment of CCX771 + STAT1si.

We further observed the change pattern of sub-G1 cells, aggressive potency, and DNA damaging extent in CRPC C4-2B cells under different treatment conditions to analyze the specific biological effects of the combination treatment of ENZ + JAK2/STAT1si that might result from the suppression of JAK/STAT-mediated signaling. As shown in Figures 4A–D, FACS confirmed that JAK2si and STAT1si could remarkably increase the percentage of sub-G1 cells compared with the control condition in C4-2B cells (JAK2si: *p* = 2.85E-06; STAT1si: *p* = 4.29E-08). Single-agent CCX771 and ENZ treatment also increased the percentage of sub-G1 cells compared with control treatment in C4-2B cells (CCX771: *p* = 1.62E-05; ENZ: 8.46E-09). The combination treatment of ENZ + JAK2si showed a significantly superior effect compared with single-agent JAK2si treatment (*p* = 3.84E-08), single-agent CCX771 treatment (*p* = 4.28E-08), single-agent ENZ treatment (*p* = 9.91E-07), and the combination treatment of CCX771 + JAK2si (*p* = 1.54E-06). The combination treatment of ENZ + STAT1si also showed a much better effect compared with single-agent STAT1si treatment (*p* = 5.92E-09), single-agent CCX771 treatment (*p* = 4.21E-09), single-agent ENZ treatment (*p* = 2.01E-08), and the combination treatment of CCX771 + STAT1si (*p* = 1.20E-07).

As displayed in Figures 4E–H, the wound-healing assay showed that C4-2B cells treated with JAK2si or STAT1si showed a significant reduction in cell migration compared with control conditions (JAK2si: *p* = 0.0371; STAT1si: *p* = 0.0031), whereas the combination treatment of ENZ + JAK2si displayed a greater effect compared with single-agent JAK2si treatment (*p* = 0.0088) and the combination treatment of CCX771 + JAK2si (*p* = 0.0379). However, a significant difference was not observed between the combination treatment of ENZ + JAK2si and single-agent ENZ/CCX771 treatment. In addition, the combination treatment of ENZ + STAT1si showed a similar suppressing effect on cell migration compared with single-agent STAT1si treatment (*p* = 0.0043), single-agent CCX771 treatment (*p* = 0.0470), single-agent ENZ treatment (*p* = 0.0495), and the combination treatment of CCX771 + STAT1si (*p* = 0.0483).

As presented in Figures 4I,J, ELISA showed that JAK2si or STAT1si treatment in C4-2B cells significantly resulted in an increase in the number of DNA fragments compared with the control condition (JAK2si: *p* = 1.20E-05; STAT1si: *p* = 5.92E-06). More importantly, the combination treatment of ENZ + JAK2si exhibited much stronger induction of DNA damage compared with single-agent JAK2si treatment (*p* = 0.0002), single-agent CCX771 treatment (*p* = 0.0006), single-agent ENZ treatment (*p* = 0.0117), and the combination treatment of CCX771 + JAK2si (*p* = 0.0325). ENZ + STAT1si also showed a better treatment effect compared with single-agent STAT1si treatment (*p* = 1.27E-05), single-agent CCX771 treatment (*p* = 4.46E-07), single-agent ENZ treatment (*p* = 4.62E-05), and the combination treatment of CCX771 + STAT1si (*p* = 0.0002). These data demonstrated that the combination treatment targeting JAK2/STAT1 and AR displayed a much stronger effect of inducing apoptosis and repressing migration in CRPC cells.

### ENZ + FLUD Strategy Synergistically Inhibited the Growth of CRPC Xenografts

**FIGURE 5:**
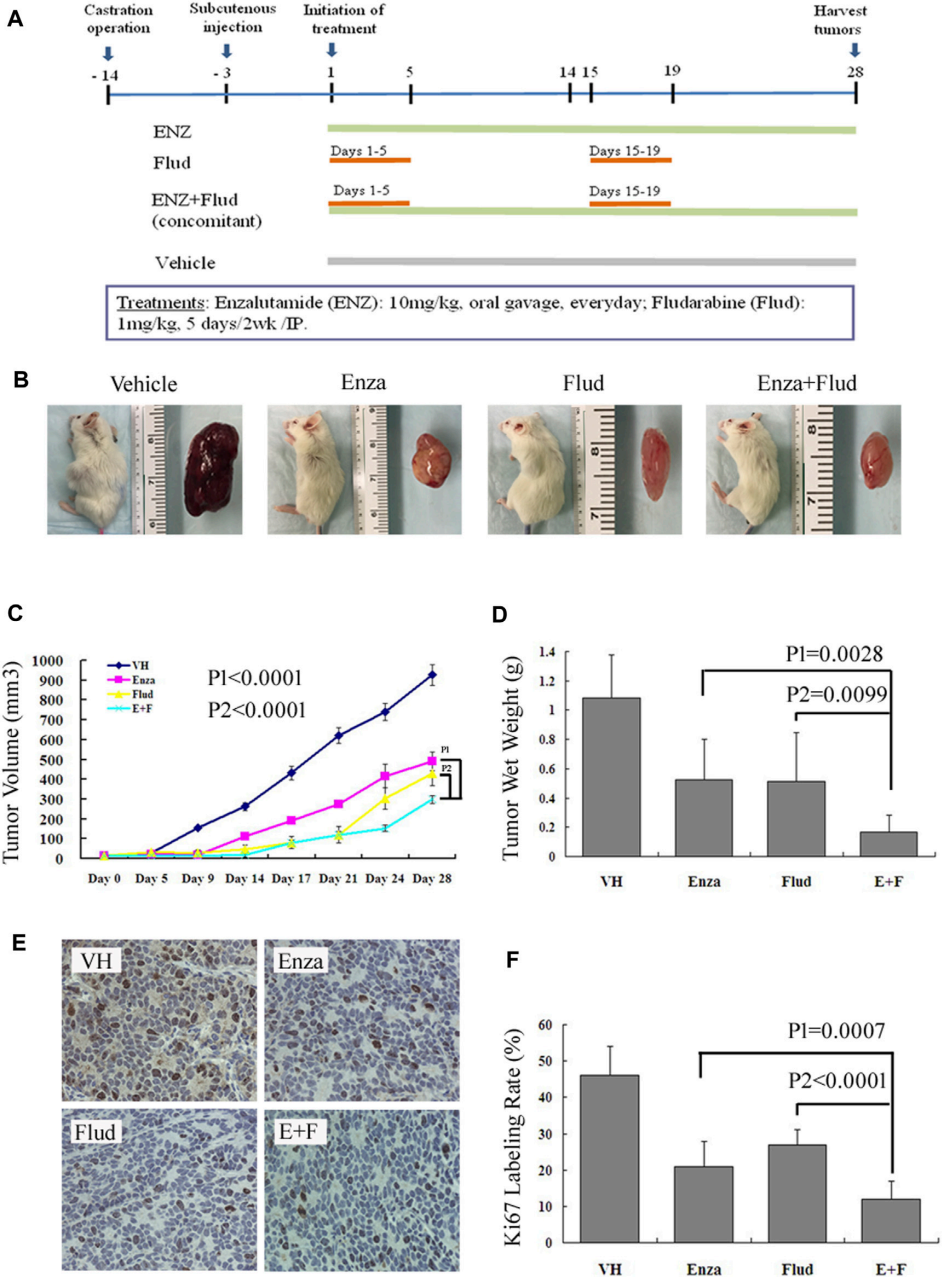
ENZ + FLUD strategy synergistically inhibited the growth of CRPC xenografts. **(A)** Drug protocol of each treatment group. **(B)** Representative C4-2B subcutaneous tumor of each treatment group. **(C)** Tumor volume measurements using calipers every 3–5 days. **(D)** Wet tumor weight at the end of 28 days of treatment. **(E)** Immunohistochemistry of Ki67 in each treatment group. **(F)** Ratio of Ki67-positive cells in each treatment group.

We used an AR-positive, androgen-independent, C4-2B subcutaneous CRPC model to further testify our hypothesis that the combination treatment of ENZ + JAK2/STAT1 was a promising therapy for CRPC. We treated mice bearing C4-2B subcutaneous tumors with ENZ alone, FLUD alone, and a combination of ENZ + FLUD and monitored their tumor progression following the protocol shown in Figure 5A.

## Discussion

The AR is an extremely important regulator that drives the progression of CRPC depending on not only self-transcriptional activity but also other signaling pathways, such as Jagged1/Notch1 pathway (Wang et al., 2010), CXCR4–CXCR7 dimer (Sun et al., 2010), EGFR/Ras/ERK (Singh and Lokeshwar, 2011), EGFR/PI3K/Akt (Mittal et al., 2009), and C-Myc (Karanika et al., 2015). The crucial step of ENZ treatment is to block the binding of androgen and AR to the maximum extent in patients with CRPC. It can suppress the transcriptional activity of the AR pathway and achieve the therapeutic purpose of promoting apoptosis and destroying DNA repair. Therefore, ENZ resistance strongly suggests the presence of alternative signaling pathways beside the AR pathway to elicit and maintain the drug-resistant progression of CRPC.

The androgen receptor shear variant AR-v7 is the first drug-resistant gene confirmed in ENZ-resistant CRPC cells. After AR-v7 was knocked out, the cell proliferation and growth were remarkably inhibited (Li et al., 2013). Clinical research further demonstrated that if tumor cells of patients with CRPC positively expressed AR-v7 (Antonarakis et al., 2014), these patients would be resistant to ENZ treatment. Therefore, AR-v7 might be a breakthrough target for ENZ resistance. In addition, our previous study also demonstrated one possible ENZ-resistance–related AR bypass pathway in which the functional role of the CXCR4–CXCR7 heterodimer shifted from CXCR4-driven to CXCR7-driven after ENZ treatment (Luo et al., 2018). However, the downstream signal transduction process of the CXCR7 pathway is still unclear.

Truncated AR splice variants (AR-Vs) are mainly due to aberrant RNA splicing intragenic and DNA rearrangement (Zhu and Luo, 2020a), resulting in the loss of the ligand-binding domain (LBD) of the AR protein. The absence of LBD makes the AR protein lose the drug-binding domain, further leading to the drug resistance of AR (Kanayama et al., 2021). Several studies attempted to explore the details of how ARv7 activated the downstream survival signal. The genomics of three ENZ-resistant CRPC PDX models, which were derived from the biopsy of skin metastasis, liver metastasis, and primary cancer, were comparatively analyzed. The results showed no consistent requirement for mutations in TP53, RB1, BRCA2, PIK3CA, or MSH2, as well as the expression of SOX2 or ERG, while all drug-resistant PDX tissues lacked PTEN expression (Zhu et al., 2020b). Additionally, enzalutamide resistance requires both AR-FL expression with at least a fiftyfold increase and AR-V7 protein expression at least sevenfold to eightfold higher than AR-FL expression in the normal prostate epithelium (Zhu et al., 2020b). Another study also involved genetic analyses in three 22RV1-based models constructed by the concomitant treatment of ADT with ABI, ENZ, and their combination on 22RV1 cells. The final results in these drug-resistant cellular models confirmed that the expression of several AR target genes, such as FKBP5, PMEPA1, and TMPRSS2, significantly increased except for the general increase in the expression of AR-FL and AR-v7 (Simon et al., 2021). Our previous research screened four genes from ENZ-resistant C4-2B cells, which were CCNA2, CKAP2L, NCAPG, and NUSAP1, and confirmed that the protein levels of these four genes also remained higher in ENZ-resistant xenograft tumor tissues (Feng et al., 2021a; Feng et al., 2021b). Although several genes with significant expression variation have been screened in different ENZ-resistant models, how these genes participate in the growth of CRPC cells is still unclear. In a previous study, we found that, after the AR was inhibited by ENZ, the activity of the TopBP1–ATR–Chk1 signaling pathway increased, resulting in the enhancement of the DDR ability of cells, which weakened the therapeutic effect of ENZ. The combination treatment of AR + ENZ and Chk1 inhibition with AZD7762 demonstrated synergistic effects with regard to decreased TopBP1–ATR–Chk1 signaling and markedly increased ATM phosphorylation and apoptosis (Karanika et al., 2017). The present study did not verify the underlying correlation between the JAK2/STAT1 pathway and the TopBP1–ATR–Chk1/DDR pathway. However, we found that the inhibition of JAK2/STAT1 activity indeed promoted apoptosis and reduced DDR. Therefore, we think that a cross-regulation mechanism exists between these two pathways.

Available evidence indicated that the activation of the JAK/STAT pathway was involved in the oncogenesis and progression of PCa. Once stimuli acted on growth factors or cytokine receptors, the autophosphorylation of one of the Janus kinases, usually JAK2, occurred. Consequently, JAK2 in turn phosphorylated a STAT family member. In particular, STAT3 is generally considered an oncogene that promotes cell survival, proliferation, motility, angiogenesis, immune tolerance, and chemotherapy resistance (Kroon et al., 2013; Ham et al., 2019; Olender et al., 2019). It is not only overexpressed in pathology specimens from prostatectomy (Barton et al., 2004) but also activated in CRPC cell lines (Agarwal et al., 2007). The upregulation of the IL-6/JAK/STAT3 cascade may contribute to promoting cell growth and survival in CRPC independent of the AR (Lou et al., 2000; Lee et al., 2003; Jia et al., 2004; Tam et al., 2007). Clinical research also found that the expression levels of phosphorylated JAK and phosphorylated STAT both positively correlated with the Gleason score and clinical stage of patients with PCa but had a negative correlation with the recurrence-free survival rates (Liu et al., 2012). Once the activation of STAT3 was inhibited, significant apoptosis could be induced in CRPC cells (Agarwal et al., 2007; Kroon et al., 2013). These reports suggested that P-STAT3 was essential for cell invasion, metastasis, and progression in patients with CRPC, especially after ADT resistance.

However, our present study demonstrated that STAT1 worked as the main signal transducer in CXCR7-driven CRPC progression. STAT1 always plays opposite roles to STAT3 in tumorigenesis and mostly triggers antiproliferative and pro-apoptotic responses while enhancing anti-tumor immunity (Huang et al., 2000; Stephanou and Latchman, 2003; Olbrich and Freeman, 2018). We found that the activation of STAT1 displayed the anomalous role of tumor-promoting factors. After the targeted inhibition of STAT1 activity, the potency of cellular survival and migration remarkably decreased, and the extent of DNA damage response and apoptosis significantly increased. It may be related to the imbalance of reciprocal regulation between STAT1 and STAT3 (Avalle et al., 2012). STAT1 was derepressed after ENZ treatment (Eiro et al., 2017). In addition, the perturbation in their phosphorylation levels may re-direct cytokine/growth factor signals from proliferative to apoptotic, or from inflammatory to anti-inflammatory (Costa-Pereira et al., 2002; Maritano et al., 2004; Shim et al., 2009; Schiavone et al., 2011; Souissi et al., 2011; Avalle et al., 2012). Several research studies suggested that the functional role of STAT1 in CRPC progression also depended on other factors, such as exon deletion (Magor et al., 2016), DNA methylation status (Dellafiore et al., 2020), different JAK family proteins (Coricello et al., 2020), and CXCR4–CXCR7 activating imbalance (Tomchuck et al., 2012; Lim et al., 2018).

Although abiraterone (ABI) does not directly antagonize AR transcriptional activity, it can maximally limit androgen synthesis, reduce the stimulating effect of androgen on AR transcriptional activity, and achieve the therapeutic purpose. Therefore, the mechanism of ENZ resistance represented by the activation of the AR bypass pathway may also be the potential mechanism of ABI resistance. Systematic evaluation of a number of clinical research studies also confirmed that the proportion of ARv7-positive patients with CRPC increased significantly after receiving new hormone therapy (NHT) (Wang et al., 2020a). This suggested that ARv7 might be involved in the cross-resistance between ENZ and ABI. Although ARv7-positive patients showed much worse treatment responses to NHT (Armstrong et al., 2020; Wang et al., 2020a; Wang et al., 2020b), the clinical data analysis demonstrated that these patients could significantly benefit from taxane chemotherapy, which was possibly related to the higher activity of the E2F1/AR3 feed-forward loop (Xu et al., 2020). The CXCR7–JAK2/STAT1 signaling pathway, as an independent AR bypass pathway, may also participate in resistance to androgen target therapy. Loss-of-function mutations in BRCA1/2 lead to a deficiency in the DNA damage repairing pathway called homologous recombination, which could render cancer cells exquisitely vulnerable to the PARP inhibitor (olaparib, OLA). However, BRCA mutations are present in only ∼20% of patients with PCa, and therefore, OLA failed to achieve the satisfied effect. Our previous study (Li et al., 2017) demonstrated that CRPC cells showed increased expression of a set of HR-associated genes, including BRCA1, RAD54L, and RMI2. Although androgen-targeted therapy is not typically effective in patients with CRPC, ENZ could significantly suppress the expression of these HR genes in CRPC cells, thus creating HR deficiency and BRCA function mutation. Pharmaceutical induction of BRCA mutation could expand the use of PARP inhibitors. Therefore, the ENZ-resistant process may synchronize with the OLA-sensitive process. The “lead-in” treatment strategy of ENZ followed by OLA may be much more effective for patients with CRPC.

Exploring the details of the AR bypass pathway may help not only clarify the ENZ-resistant mechanism but also improve the treatment sensitivity of other NHT drugs, PARP inhibitors, and chemotherapy. The present study demonstrated that the JAK2/STAT1 signaling pathway was closely involved in the ENZ resistance process induced by CXCR7 derepression. Also, the inhibition of JAK2/STAT1 could notably improve the ENZ treatment effect and lead to the downregulation of C-Myc, decreasing motility and proliferation and increasing apoptosis and DNA damage. Target inhibition of the CXCR7–JAK2/STAT1–C-Myc signaling pathway may be an important and effective strategy to overcome ENZ resistance in patients with CRPC.

## Data Availability

The raw data supporting the conclusions of this article will be made available by the authors, without undue reservation.
